# Sleep smart—optimizing sleep for declarative learning and memory

**DOI:** 10.3389/fpsyg.2015.00622

**Published:** 2015-05-12

**Authors:** Gordon B. Feld, Susanne Diekelmann

**Affiliations:** Institute for Medical Psychology and Behavioral Neurobiology, University of TübingenTübingen, Germany

**Keywords:** sleep, memory, learning, consolidation, applied research

## Abstract

The last decade has witnessed a spurt of new publications documenting sleep's essential contribution to the brains ability to form lasting memories. For the declarative memory domain, slow wave sleep (the deepest sleep stage) has the greatest beneficial effect on the consolidation of memories acquired during preceding wakefulness. The finding that newly encoded memories become reactivated during subsequent sleep fostered the idea that reactivation leads to the strengthening and transformation of the memory trace. According to the active system consolidation account, trace reactivation leads to the redistribution of the transient memory representations from the hippocampus to the long-lasting knowledge networks of the cortex. Apart from consolidating previously learned information, sleep also facilitates the encoding of new memories after sleep, which probably relies on the renormalization of synaptic weights during sleep as suggested by the synaptic homeostasis theory. During wakefulness overshooting potentiation causes an imbalance in synaptic weights that is countered by synaptic downscaling during subsequent sleep. This review briefly introduces the basic concepts and central findings of the research on sleep and memory, and discusses implications of this lab-based work for everyday applications to make the best possible use of sleep's beneficial effect on learning and memory.

## Introduction

**Figure 1:**
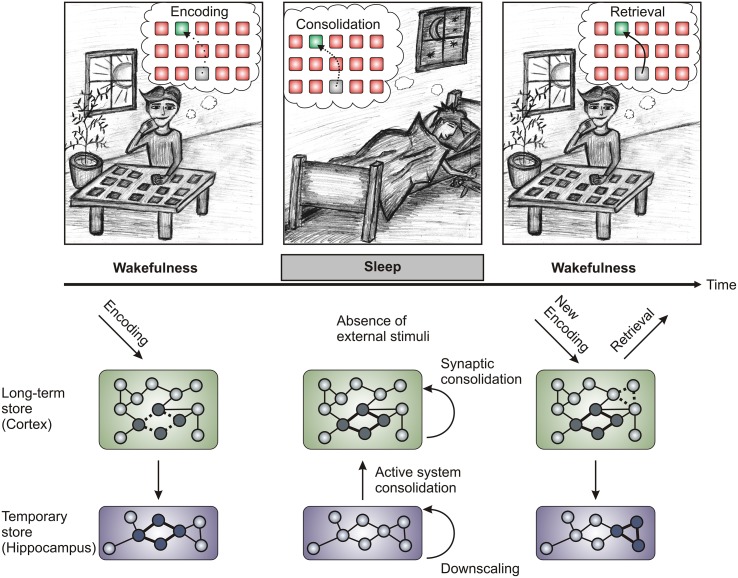
**Schematic of the proposed memory processes. Upper panel:** Memory processes can be divided into three steps (Encoding, Consolidation, and Retrieval). Encoding is the process of information uptake during learning (in this case of card pair locations of a concentration game) and is most effective during wakefulness, while retrieval is the process of recalling the memorized content at a later time point. Consolidation takes place after encoding and is necessary to transform the initially labile traces into enduring representations. During sleep after learning, sleep-dependent memory consolidation strengthens the acquired memory traces by reactivating (“replaying”) them. **Lower panel**: During wake encoding, information is taken up by the sense organs and flows to the hippocampus via the association cortices. In the hippocampus a trace is formed fast but only for temporary storage (thick lines in the hippocampus represent the hippocampal trace, dashed lines in the cortex represent the corresponding cortical trace). During slow wave sleep the traces in the hippocampus are reactivated leading to their integration into the long-term store of the cortex (active system consolidation), where they are strengthened through synaptic consolidation (the consolidated trace is represented by thick lines in the cortex). Additionally, processes of synaptic downscaling during sleep (shown as the hippocampal trace vanishing) lead to the preparation of the brain to encode new information during the next wake phase (again depicted by thick and dashed lines in hippocampus and cortex, respectively). While the synaptic downscaling hypothesis has been most rigorously applied to cortical learning tasks (Tononi and Cirelli, 2014), recent research has provided evidence for downscaling in the hippocampus during REM sleep (Grosmark et al., 2012). Processes of local synaptic strengthening may work in concert with global downscaling processes to provide reliable and efficient memory traces (Born and Feld, 2012). **Bottom panel** is adapted from Diekelmann and Born (2010).

**Figure 2:**
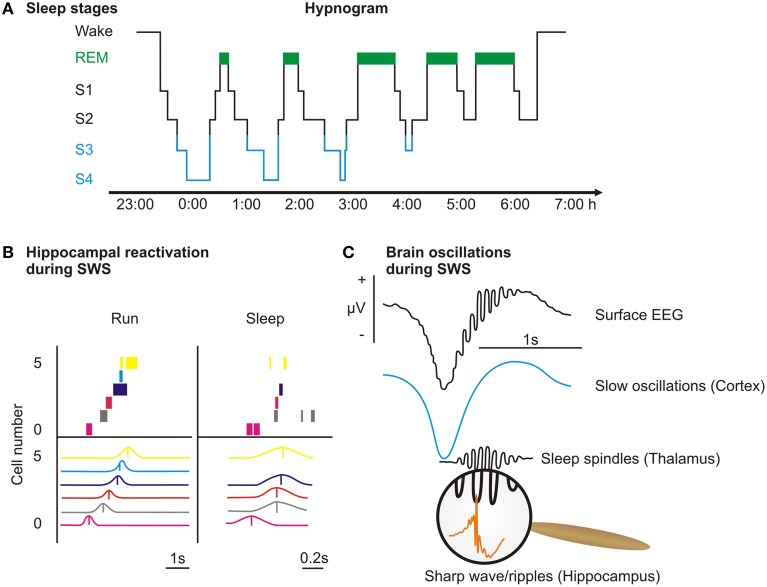
**Overview over sleep stages. (A)** Sleep, defined as a readily reversible state of reduced responsiveness to the environment, is a phenomenon reported in all animals—from humans to flies and mollusks—that have been systematically examined so far (Borbély and Achermann, 2000; Huber et al., 2004b; Cirelli and Tononi, 2008; Vorster et al., 2014). Human sleep is traditionally dichotomized into rapid-eye-movement (REM) sleep and non-rapid-eye-movement (NonREM) sleep and prototypically consists of 90 min cycles, during which NonREM sleep and REM sleep alternate. REM sleep (green) is characterized by rapid eye movements, a mixed frequency EEG and an inhibition of muscle tone. NonREM sleep can be further subdivided into sleep stages 1–4 (S1–S4), which resemble the depth of sleep, inasmuch as responsiveness to external stimuli is minimal in sleep stage 4. S1 is a transitory stage that only makes up a small amount of a night's sleep (<10%). S2 is determined by the occurrence of sleep spindles (waxing and waning activity of 10–15 Hz) and K-complexes (a sharp negative high-amplitude deflexion followed by a slower positive wave). S3 and S4 combine to slow wave sleep (SWS, blue) that is characterized by large amounts (>20%) of slow wave activity (0.5–4 Hz) in the EEG. **(B)** In rats, place cells express a specific firing pattern dependent on the rat's location within its environment. When running through a maze (Run) this leads to a sequential firing pattern that represents the sequence of locations the rat passes in the maze (each row represent one cell, the upper panel shows spikes across time during one lap, the lower panel shows averaged activity). During slow wave sleep (Sleep) after training on an alternation task in the maze the firing sequence is replayed in a time-compressed manner (adapted from Ji and Wilson, 2007). This and other similar experiments are evidence that representations encoded during wakefulness are replayed during subsequent sleep. **(C)** Recent evidence suggests that the hallmark oscillation of SWS, the sleep slow oscillation (<1 Hz), which is generated in cortical areas, coordinates replay in the hippocampus (accompanied by sharp wave/ripple activity generated in the hippocampus) and plasticity-promoting spindle activity (generated in the thalamus). The coordination of these oscillations enables the gradual transfer of the transient memory representations from the hippocampus to their long-term storage sites within the cortex. The upper trace depicts the unfiltered surface EEG and below, the surface EEG filtered in the slow oscillation and the spindle band are shown (spindles typically occur in the down-to-up transition of the slow oscillation). The inset shows sharp-wave/ripple activity, which is typically nested in the troughs of spindles.

Our modern society has formed an ill-advised but strong belief that sleep is an annoying habit that should be kept at a strict minimum to enhance productivity. However, recent research indicates that sleep's function goes beyond rest and replenishment (in itself a good reason to sleep) and constitutes a state of active offline information processing essential to the appropriate functioning of learning and memory. Memory is established in three stages; new memories are initially acquired (encoding), become strengthened and reorganized (consolidation), and are finally recalled (retrieval; Figure 1 upper panel). In 1924, Jenkins and Dallenbach expanded on Ebbinghaus (1885) studies of forgetting curves and reported that sleep after encoding of nonsense syllables supports memory consolidation, inasmuch as it reduces forgetting compared to an interval containing only wakefulness (Jenkins and Dallenbach, 1924). This line of research was revisited several times during the last century (Ekstrand, 1967; Yaroush et al., 1971; Barrett and Ekstrand, 1972; Fowler et al., 1973; Benson and Feinberg, 1975; Ekstrand et al., 1977) and progress was aided substantially by the identification of sleep stages with distinct psychophysiological properties (Figure 2; Aserinsky and Kleitman, 1953; Dement and Kleitman, 1957). During the last two decades, interest in the topic has re-emerged and we have witnessed an upsurge of publications dissecting sleep's role for memory (for a comprehensive review see Rasch and Born, 2013). One of the most widely studied models of sleep's role for memory consolidation, according to the active system consolidation theory, is that of an active neuronal replay of memory representations during slow wave sleep, strengthening memory traces encoded during preceding wakefulness (Figure 1 upper panel; Diekelmann and Born, 2010; Oudiette and Paller, 2013). This theory has recently received support by findings showing that sleep following learning increases synaptic spines specifically associated with prior learning experience (Euston and Steenland, 2014; Yang et al., 2014). Apart from the consolidating effect, sleep has an additional function in the restoration of learning capabilities (Van Der Werf et al., 2009; Antonenko et al., 2013), which may also be linked to the downscaling of synapses that were potentiated during prior wakefulness (Tononi and Cirelli, 2003, 2006, 2014). Of note, this review does not touch on the subject of learning during sleep, which to the best of our knowledge has not been shown in the declarative memory domain. Empirical work suggests that encoding during sleep in humans, if at all, is only possible for conditioning (Ikeda and Morotomi, 1996; Arzi et al., 2012, 2014; Cox et al., 2014).

While we are only starting to understand the neuronal mechanisms that underlie these processes, the following sections give a brief overview of some of the most persuasive findings in an attempt to translate them into behavioral guidelines for optimizing memory function by sleep. Importantly, rather than giving a state of the art overview of all findings and developments in the rapidly expanding field of sleep and memory (for recent comprehensive reviews see, Diekelmann and Born, 2010; Lewis and Durrant, 2011; Saletin and Walker, 2012; Abel et al., 2013; Inostroza and Born, 2013; Oudiette and Paller, 2013; Prince and Abel, 2013; Rasch and Born, 2013; Stickgold and Walker, 2013), this review focuses on the most established findings to aid their adaptation into education, therapeutic practice and performance improvement in the elderly. Additionally, we hope to encourage an increase in applied research of sleep and memory, which is still in its beginnings.

## Consolidating memory during sleep

### Active system consolidation during sleep

The theory of active system consolidation can explain how sleep is able to strengthen, transform and redistribute the information that was encoded during prior wakefulness (Figure 1 lower panel; Diekelmann and Born, 2010; Rasch and Born, 2013). During wakefulness new information is encoded in parallel into the hippocampus, which serves as a fast-learning temporary store, and into distributed cortical areas that represent the slow-learning long-term store. Because the cortex takes longer to form direct connections between single elements of a memory, the hippocampus initially acts as a hub to bind the cortical representations (Winocur et al., 2010; Battaglia et al., 2011). During sleep, the new memory representations become re-activated in the hippocampus as well as in the cortex. The hippocampus thereby serves as a trainer for the cortical long-term store by reactivating the cortical representations repeatedly so that the cortical connections become strengthened and eventually become independent of the hippocampus.

### Reactivation of memory traces during sleep

Such a neuronal “replay” of learning-related activity during sleep was first observed in rats. After a spatial learning experience that activated specific place cells in the hippocampus, those cells that had fired together during learning displayed correlated firing again during subsequent sleep (Wilson and McNaughton, 1994). Importantly, replay in the hippocampus follows the same sequence as during wakefulness and is also coordinated with replay in the cortex (Figure 2B; Skaggs and McNaughton, 1996; Ji and Wilson, 2007). Other brain regions show replay of learning-related neuronal activity during sleep as well, such as motor cortex, striatum, and thalamus (Ribeiro et al., 2004; Lansink et al., 2008, 2009; Gulati et al., 2014; Harris, 2014). Signs of reactivation were also found in humans using brain imaging techniques such as functional magnetic resonance imaging (fMRI). In these studies, participants learned tasks that activated specific brain regions and similar activation patterns were re-expressed during subsequent sleep (Maquet et al., 2000; Peigneux et al., 2004). Memory reactivations are accompanied by high-frequency oscillatory events (ripples, 100–200 Hz) in the local field potential of the hippocampus that together with sharp-waves form sharp-wave/ripple complexes (Figure 2C, Buzsaki, 1986, 1989; Nadasdy et al., 1999). The sleep slow oscillations (large amplitude waves <1 Hz in the EEG) that dominate slow wave sleep are generated in the cortex and synchronize these memory reactivations in the hippocampus (Sirota et al., 2003; Buzsaki and Draguhn, 2004; Molle and Born, 2011). This synchronization effects that the reactivated memory information reaches the cortex together with sleep spindles (10–15 Hz oscillations, Mölle et al., 2011; Bergmann et al., 2012). The plasticity enhancing effect of spindles in the cortex facilitates the integration of the new memories into the long term store (Ribeiro et al., 2007) benefiting the recall of these memories at a later point. These oscillations and the associated memory reactivations are partly dependent on the specific constellation of hormones and neurotransmitters during sleep, such as the low cholinergic tone during slow wave sleep (Hasselmo, 1999; Gais and Born, 2004), which allows information to flow from the hippocampus to the neocortex. While there are also accounts of memory reactivation during wakefulness and REM sleep, it seems to be the specific combination of the oscillatory and neuromodulatory environment found during slow wave sleep that enables consolidation through reactivation in the hippocampus and cortex (Diekelmann and Born, 2010).

### Reorganization of memory and extraction of invariant features

Beyond the strengthening of memories, sleep also reorganizes and transforms memories. This reorganization of memory can facilitate generalization and the abstraction of the gist, i.e., invariant features or rules, from encoded information (Gomez et al., 2006; Durrant et al., 2011; Lewis and Durrant, 2011; Inostroza and Born, 2013). Sleep can even help to gain insight and find new solutions to problems. Wagner et al. (2004) used a number reduction task that afforded participants to perform a series of numerical transformations. Unbeknown to the participants there was a hidden rule in the task. As soon as they discovered this rule they could use a short cut to solve the task. Almost three times more participants in the sleep condition gained insight into this hidden rule compared to the wake condition. Further, evidence for a reorganization of memory during sleep comes from studies in which false memories were provoked by asking participants to learn lists of words that all pertained to an associated word (the gist word) that was not on the list. More gist words were falsely assigned as true memories by those participants that slept during the retention interval compared to those who remained awake (Payne et al., 2009; Diekelmann et al., 2010), suggesting that sleep helped extracting the general meaning of the word lists.

## Restoration of learning capacity during sleep

### Restoring synaptic homeostasis during sleep

Next to the idea of active system consolidation, the most influential theory of sleep's function for memory is the synaptic homeostasis hypothesis (Tononi and Cirelli, 2003, 2006, 2014). According to this hypothesis, the widespread synaptic potentiation occurring at encoding of information during wakefulness leads to increased demands of space and energy in the brain. If this development remained unchecked the brain would shortly reach the limits of its encoding and/or upkeep capabilities. Sleep is essential to renormalize synaptic weights (termed “synaptic downscaling”) and has therefore been suggested to be “the price we pay for plasticity” (Cirelli and Tononi, 2008; Tononi and Cirelli, 2014). Indeed, overall synaptic spines as well as markers for synaptic potentiation increase across periods of wakefulness and decrease during periods of sleep (Vyazovskiy et al., 2008; Maret et al., 2011). Computer simulations have established the theoretical effectiveness of slow wave activity (0.5–4 Hz) to renormalize synapses (Hill and Tononi, 2005; Esser et al., 2007; Sullivan and de Sa, 2008), and potentiating synapses by extended learning (Huber et al., 2004a; Schmidt et al., 2006) or by transcranial magnetic stimulation (Huber et al., 2007, 2008) induces local increases of slow wave activity. Conversely, immobilizing a human's arm reduces slow wave activity (Huber et al., 2006) and slow wave sleep deprivation impairs visuo-motor learning (Hill et al., 2008; Aeschbach, 2009; Landsness et al., 2009). In correspondence with the general finding that slow wave activity is homeostatically regulated (Borbély and Achermann, 2000) this line of research indicates that slow wave activity may play a major role for synaptic homeostasis (Massimini et al., 2009; Vassalli and Dijk, 2009). However, it has also been shown that learning increases sleep spindles (Gais et al., 2002), and since the synaptic homeostasis theory makes no explicit claims as to the specific mechanisms involved (Tononi and Cirelli, 2012), their exact nature as well as their association with specific sleep stages remain to be determined (Born and Feld, 2012; Chauvette et al., 2012; Grosmark et al., 2012).

### Improved learning after sleep

A direct consequence of synaptic downscaling during sleep can be seen in the improvement of learning after a period containing sleep compared to a period containing only wakefulness (McDermott et al., 2003). In a nap study, Mander and colleagues tested their participants' ability to learn new information twice in 1 day, once at noon and a second time in the evening. One group of subjects was allowed to take a nap in between the two sessions and another group stayed awake. As expected, encoding ability deteriorated across the day in the wake subjects, while subjects who took a nap even slightly improved encoding performance at the second session (Mander et al., 2011). Impairments in encoding ability were also observed in participants who were sleep-deprived for one night, with these impairments being associated with reduced hippocampal activation (Yoo et al., 2007). In line with the suggestion of the synaptic homeostasis hypothesis that slow waves might be responsible for synaptic downscaling, it was shown that suppressing slow wave sleep in elderly participants reduced their ability to encode new information the next morning (Van Der Werf et al., 2009).

## Optimizing sleep's beneficial influence on memory

### Application of sleep's natural effects on memory

Although a considerable number of findings suggest that sleep plays a pivotal role for the normal functioning of human memory, research on the application of these effects of sleep on memory is still at its beginning. The translation of the basic science findings into real world contexts can not only help improve our learning and memory capacities in everyday life but is also a promising avenue for possible applications in aging, clinical settings, and education. Based on the available evidence, the following sections offer potential strategies of optimizing sleep's beneficial influence on memory in applied contexts.

#### Timing and amount of sleep

The timing of sleep has been shown to influence sleep's effect on memory, inasmuch as a shorter interval between sleep and learning of declarative tasks enhances the effect of sleep on memory (Gais et al., 2006; Talamini et al., 2008; Payne et al., 2012b). Comparing retention intervals that differed with respect to the timing of sleep, Gais et al. (2006) found that sleep that follows within 3 h after encoding is more beneficial to the consolidation of English-German vocabulary than sleep following after 10 h. Thus, to make the best use of sleep's memory improving effect, sleep should follow encoding within a few hours or, alternatively, the contents that were learned during the day could be rehearsed shortly before bedtime. With regard to the amount of sleep that is necessary to boost memory, several studies have shown that declarative memory even benefits from rather short periods of sleep. 3-h episodes of sleep during the first half of the night are sufficient to prompt the beneficial effect of sleep on declarative memory (Yaroush et al., 1971; Barrett and Ekstrand, 1972; Fowler et al., 1973; Plihal and Born, 1997; Tucker and Fishbein, 2009) and even naps can be used to improve memory, albeit, in a more dose-dependent manner (Lahl et al., 2008). Although some studies found memory improvements of very short naps of only a few minutes (Lahl et al., 2008), longer periods of 60–90 min of sleep provide better outcomes (Diekelmann et al., 2012). This offers the opportunity to time short naps after intensive periods of learning to benefit consolidation and subsequent learning. However, even though short amounts of sleep can help the initial consolidation of new memories, naps cannot replace a good night's sleep but may rather be introduced in addition to nocturnal sleep. Some types of memories even require a whole night of sleep, including the cyclic occurrence of different sleep stages, to become optimally consolidated (Diekelmann and Born, 2010).

#### The role of relevance and reward

Memory consolidation during sleep is selective in preferentially facilitating those memories that are in some way relevant for the future. Wilhelm et al. (2011) showed that merely the information that memory contents will have to be recalled on the next day can decide its access to sleep-dependent consolidation. In this study participants learned a declarative word pair association task in the evening. After encoding, only one of two groups was informed that they would have to retrieve the word pairs the next morning. While the informed group showed the expected improvement in retention performance after sleep, the uninformed group did not and was on par with an informed group that did not sleep during the retention interval. This effect is confirmed by sleep's enhancement of prospective memories of tasks to be performed in the future (Scullin and McDaniel, 2010; Diekelmann et al., 2013a,b). Similar findings were observed when subjects anticipated a monetary reward for performing well in a procedural finger sequence tapping task (Fischer and Born, 2009). Emotionality of the learning content is a further feature that signals relevance for the individual and leads to stronger effects of sleep-dependent memory consolidation (Wagner et al., 2001; Sterpenich et al., 2007, 2009; Payne et al., 2008, 2012a; Groch et al., 2013, 2014). However, there seem to be conflicting mechanisms of REM sleep for consolidating emotional content and SWS for declarative content in some tasks (Payne et al., 2008; Groch et al., 2014). To optimize the beneficial effect of sleep on memory, the relevance of the learning material should be very evident to the learner and might be increased by additional incentives.

#### Sleep and memory in children and students

Sleep also benefits the learning of declarative tasks such as word pair associations and object locations in children (Wilhelm et al., 2008). A recent study in preschoolers shows that learning new words from storybooks that are read to them is increased in children that are allowed to nap after hearing the story (Williams and Horst, 2014). Even infants of 6–16 months learn new word meanings or novel actions better if they nap during the retention interval (Friedrich et al., 2015; Seehagen et al., 2015). For the extraction of explicit knowledge, sleep might even be more beneficial in children than in adults. Wilhelm and colleagues trained adults and 8–11-year-old children to press a specific sequence of buttons on a button-box (Wilhelm et al., 2013). None of the participants were aware of the underlying sequence of button presses during training before sleep. While both children and adults gained more explicit awareness of the sequence after sleep compared to a wake interval, children clearly outperformed the adults after sleep with almost all children being able to explicitly generate the entire 8-element sequence. Interestingly, this beneficial effect of sleep on knowledge may even extend into highly demanding education such as medical school (Ahrberg et al., 2012; Genzel et al., 2013). In a questionnaire study, sleep behavior (especially during the pre-exam period), together with the results in the pre-clinical board exam, turned out as the strongest predictor for the final grade in the medical exam (Genzel et al., 2013). Improving sleep in children and students might therefore be a promising target to improve school performance (Ribeiro and Stickgold, 2014).

#### Sleep and memory in the elderly

Older adults typically show a dramatic reduction in the amount of slow wave sleep, with this reduction being associated with reduced sleep-dependent memory consolidation (Backhaus et al., 2007). The importance of slow wave sleep for memory consolidation in the elderly was shown in a cross-sectional study (Mander et al., 2013b). In this study young and elderly adults learned a declarative word pair task before going to sleep. Overnight retention was predicted by slow wave activity, which in turn was associated with prefrontal gray matter volume. The authors argue that the age-related reduction in gray matter volume causes the decline of slow wave generation in the brain, which in turn compromises sleep's ability to consolidate memories. In another study from the same group, it was shown that the reduction in sleep spindles observed in the elderly is predictive of impairments of new learning (of an episodic task) and hippocampal activation after sleep (Mander et al., 2013a). Importantly, in these studies it remains unclear if sleep is causal to the observed impairments. Clarifying this question will be essential to identify therapeutic targets for improving sleep and memory in the elderly.

#### Improving psychotherapy outcome with sleep

Many memory-related psychiatric disorders, such as post-traumatic stress disorder (PTSD), are associated with altered sleep profiles (Germain, 2013; Steiger et al., 2013), suggesting that sleep might play a role in the etiology of these disorders. Considering that behavioral therapy is effective in many anxiety disorders and relies on learning mechanisms, its success may be further improved by using sleep treatments. Particularly in phobias diminished extinction of conditioned fear responses is often a problem. Sleep benefits the generalization of the extinction of conditioned fear (Pace-Schott et al., 2009) and this finding has been applied to simulated exposure therapy showing that sleep can be used to consolidate and generalize extinction of spider fear responses (Pace-Schott et al., 2012). Recently, Kleim et al. (2013) were able to show that napping for 90 min after an exposure therapy session can reduce self-reported fear and negative cognition at a 1-week follow-up assessment in patients suffering from spider phobia. Hence, applying sleep as a non-invasive method might prove to be a highly useful and inexpensive tool to boost the efficiency of psychotherapy.

### Enhancing sleep's beneficial influence on memory

Apart from the knowledge about how to make best use of natural sleep for memory functions, recent research approaches have also produced a number of insights of how to enhance memory processing during sleep beyond its natural boundaries. Although there are still certain methodological and ethical restraints (for a topic specific review see, Diekelmann, 2014), memory enhancement during sleep might prove worthwhile in daily contexts as well as in clinical applications.

#### Cueing memory reactivation during sleep

Recent research suggests that memory replay can be biased by external cues presented during sleep that had been present during prior encoding (Bendor and Wilson, 2012). In their seminal paper, Rasch et al. (2007) used odor cues to trigger memory reactivation processes during sleep in humans. Participants were asked to encode locations of picture pairs shown on a computer screen (similar to the game concentration, Figure 1 upper panel) while they smelled the scent of roses. During subsequent slow wave sleep participants were re-exposed to this odor or an odorless vehicle. Those participants who had smelled the odor during learning and during slow wave sleep showed increased recall of the card locations at retrieval compared to the vehicle condition. Memory was not improved if participants received the odor only during sleep but not during prior encoding, and odor re-exposure was also not effective during wakefulness and post-learning REM sleep. Since this report, the paradigm has been extended to show that specific auditory cues associated with picture-locations (e.g. the sound “meow” associated with the picture of a cat at a specific location), likewise foster memory reactivation if presented during slow wave sleep, increasing subsequent recall performance (Rudoy et al., 2009; van Dongen et al., 2012). Additionally, it was shown that auditory cues during sleep can enhance the retention of non-declarative tasks by using a melody for cueing a sequence of button presses in a motor skill task (Antony et al., 2012). Interestingly, this effect is highly specific, inasmuch as participants only perform better on that part of the sequence that has been cued during sleep, while performance on the un-cued part is unchanged (Schonauer et al., 2014). Finally, it was demonstrated that the strengthening effect of reactivation cues for memory is specific for the sleep state and that presentation of the same cues during wakefulness can even labilize and disturb the memory trace (Diekelmann et al., 2011). Both odor cues and tones are stimuli that are easy to apply in the home environment to improve individual memory capacities. For example, students might consider using fragrance lights or aromatic lamps during learning and subsequent sleep, or re-play previously learned spoken vocabulary during sleep. Care should be taken, however, to make sure that these stimuli do not disturb sleep *per se*.

It has now been attempted to translate the memory cueing paradigm to fear conditioning tasks, which opens the possibility of enhancing efficiency of exposure therapy. In one of these studies participants who had undergone olfactory contextual fear conditioning were re-exposed to the olfactory context cue during subsequent slow wave sleep. Compared to the control condition without odor during sleep, these participants showed greater extinction of contextual fear in the next morning (Hauner et al., 2013). Likewise, subjects who were fear conditioned on a neutral tone and were re-presented this tone during slow wave sleep, showed reduced fear responses to the tone after sleep (He et al., 2014). Two similar studies in rodents, however, showed a greater fear response after re-exposure of the conditioned stimulus during sleep (Rolls et al., 2013; Barnes and Wilson, 2014). Although much more research is needed to clarify these controversial findings and to identify possible boundary conditions and adverse effects (for an opinion on the contradictory nature of these results see, Diekelmann and Born, 2015), this method bears great potential for applications in clinical contexts, e.g., for the treatment of anxiety disorders.

#### Stimulation of brain oscillations

Slow oscillations are the brain oscillations that have been most closely linked to sleep-dependent memory processing. Intensifying these slow oscillations with electrical or auditory stimulation has been shown to enhance learning and memory consolidation (Marshall et al., 2006; Ngo et al., 2013b). In the ground-breaking study by Marshall and colleagues, participants learned a word pair association task in the evening. During subsequent sleep they were subjected to trans-cranial electric stimulation (TES) at the same frequency as natural slow oscillations (~0.75 Hz). This stimulation did not only intensify slow oscillations as well as spindle activity, but also increased the efficacy of sleep to consolidate the previously learned word pairs (Marshall et al., 2006). Using the same stimulation at the same frequency (~0.75 Hz) during wakefulness increases theta activity and improves encoding of new learning material, while consolidation remains unchanged (Kirov et al., 2009). Finally, applying this slow oscillation stimulation during a daytime nap improves the encoding of new information afterward (Antonenko et al., 2013), suggesting that slow wave activity during prior sleep frees space for new learning after sleep. Even though some consumer devices for TES have become available (e.g., http://www.foc.us/, http://thebrainstimulator.net), the possibility to affect sleep's slow oscillations by auditory stimulation may offer a more elegant and less invasive approach for the bedroom (Ngo et al., 2013a). Ngo et al. timed short (50 ms) bursts of white noise on the slow oscillation up-states, which increased the amount of consecutive slow oscillations as well as spindle activity coupled to the slow oscillations. When compared to a sham condition, the stimulation improved sleep-dependent consolidation of declarative word pair memory (Ngo et al., 2013b). Such an auditory stimulation of slow oscillations could be applied in the home environment to boost memory performance with relatively little effort. Very recently it has been shown that slow wave sleep can even be enhanced by hypnosis (Cordi et al., 2014, 2015). This method may offer the possibility of enhancing sleep without recurring to invasive and potentially sleep-disturbing techniques.

#### Using drugs to enhance memory during sleep

Many neurotransmitters and hormones that are known to be implicated in learning and memory show characteristic changes across the sleep-wake cycle. It therefore stands to reason to apply certain drugs targeting specific neurotransmitter systems during sleep to enhance memory. GABA A modulators, for example, have long been known to induce sleep and have thus been used extensively to treat sleep disorders. Yet, these drugs may not induce natural sleep but rather light sleep with low amounts of slow wave sleep and REM sleep (Lancel, 1999). The GABA re-uptake inhibitor tiagabine, on the other hand, increases the occurrence of slow wave sleep, but unfortunately decreases sleep spindles and even deteriorates consolidation of a procedural motor skill task (Feld et al., 2013b). The GABA A positive modulator zolpidem has been shown to have a more beneficial profile, as it increases the amount of sleep spindles and improves memory consolidation of word pairs (Kaestner et al., 2013; Mednick et al., 2013). However, other authors do not find this effect or even observed a detrimental effect of zolpidem on declarative memory consolidation during sleep (Melendez et al., 2005; Hall-Porter et al., 2014). Recently it was shown that the NMDA receptor co-agonist d-cycloserine can enhance the consolidation of word pair associations if given before a retention interval containing sleep (Feld et al., 2013a). D-cycloserine has also been discussed as a cognitive enhancer to improve efficacy of cognitive behavior therapy to attenuate anxiety disorders (Otto et al., 2007; Hofmann et al., 2012), hence the application of d-cycloserine during retention sleep after successful exposure sessions may increase the therapeutic value of this drug. Other pharmacological treatments have likewise been shown to affect memory formation during sleep more or less beneficially, such as the noradrenaline reuptake inhibitor reboxetine (Rasch et al., 2009; Gais et al., 2011), the cytokine interleukin-6 (Benedict et al., 2009), and the dopamine D2-like receptor agonist pramipexole (Feld et al., 2014). Generally, drugs that increase sleep and memory are potentially interesting for clinical applications, but should presently not be considered for non-therapeutic sleep and memory enhancement, as the potential adverse effects remain completely unclear.

## Concluding remarks

Sleep is not idle time. Sleep is an active state during which the brain processes information acquired during the previous day and prepares itself for the demands of the next day. Long-term memory is formed during sleep by a process that strengthens memory traces, reorganizes them, and integrates them into established knowledge networks. These processes do not only store previously acquired memories but also renew the capacity for new learning after sleep. Considering this backdrop, it is alarming how society still views sleep as a luxury. Sleep is not only a necessity. Rather we should start recognizing sleep as a worthy and skillful operator that helps our brain to work at its best. This review has discussed several ways of how to make optimal use of sleep's helpful hand in learning and memory. It is quite clear that improved sleeping conditions will have a higher impact on overall productivity than increasing the mere presence at work or at the study table. The ideal strategy to acquire new information should consider that sleep is important for memory. For students it is probably not effective to skip sleep in order to have more hours to learn for a specific subject. And for finding elegant solutions to complex problems the popular proverb may offer good advice: sleep on it.

### Conflict of interest statement

The authors declare that the research was conducted in the absence of any commercial or financial relationships that could be construed as a potential conflict of interest.
